# Single-Molecule Break Junctions Based on a Perylene-Diimide Cyano-Functionalized (PDI8-CN_2_) Derivative

**DOI:** 10.1186/s11671-015-1011-3

**Published:** 2015-07-28

**Authors:** Riccardo Frisenda, Loredana Parlato, Mario Barra, Herre S.J. van der Zant, Antonio Cassinese

**Affiliations:** Kavli Institute of Nanonscience, Delft University of Technology, Lorentzweg 1, 2628 CJ Delft, The Netherlands; CNR-SPIN and Physics Department, University of Naples, Piazzale Tecchio 80, I-80125 Naples, Italy

**Keywords:** Perylene-diimide molecules, Single molecule, Break junctions, Conductance, Density functional theory

## Abstract

In this letter, we report the single-molecule conductance properties of a cyano-functionalized perylene-diimide derivative (PDI8-CN_2_) investigated with gold nano-electrodes. This molecule is of large interest for the fabrication of high-performance and air-stable n-type organic field-effect transistors. Low-bias experiments performed on mechanically controllable break junctions reveal the presence of two different values of the single-molecule conductance, which differ by about two orders of magnitudes. Up to date, this feature was never observed for other perylene-diimide compounds having alternative chemical moieties attached to the basic aromatic core. Theoretical calculations suggest that the highest single-molecule conductance value here observed, comprised between 10^−2^ and 10^−3^ G_0_, is related to a charge transport path directly linking the two cyano groups.

**Electronic supplementary material:**

The online version of this article (doi:10.1186/s11671-015-1011-3) contains supplementary material, which is available to authorized users.

## Background

Perylene-diimide (PDI) molecules are aromatic compounds, being synthesized for the first time one century ago, and then widely employed as industrial organic pigments [1]. In the second half of the 1990s, the scientific and technological interest for this chemical family was considerably renewed by the demonstration of their appealing opto-electronic properties to be exploited in the development of innovative devices [2–4]. In particular, it was demonstrated that, in reason of their excellent self-assembling properties, PDI films are capable to effectively carry charges when used as active channels of n-type (electron transporter) Organic Field-Effect Transistors (OFET) [5, 6]. In this regard, it is worth to mention that, still at present, the available number of efficient n-type organic semiconductors is significantly smaller than that of p-type (hole transporter) counterparts, posing severe limitations to the full development of complementary integrated organic circuits.

In the last 20 years, considerable research efforts have been consequently undertaken to synthesize novel PDI derivatives, displaying even better charge transport properties with the specific requirement to preserve the electrical behavior quality also under ambient conditions. Within this context, perylene-diimide molecules (PDI_CY), functionalized through the insertion of cyano (C≡N) groups in the bay molecular region, have been demonstrated to exhibit unique performances [7, 8]. Indeed, several experimental works have clarified that the cyano functionalization allows to considerably lower the LUMO levels (down to −4.5 eV) without impairing the PDI self-assembling capabilities. In the realization of n-type OFET, the LUMO level lowering provides a twofold beneficial effect: firstly, an effective charge-injection can be achieved using gold or other high work function metals, and secondly, the electron transport is made less sensitive to charge trapping mechanisms related to the interaction with ambient agents such as oxygen and humidity [4]. Moreover, when deposited in form of films by evaporation, PDI_CY molecules follow a basic layer-by-layer growth mode on various surfaces of relevant technological interest, such as for example SiO_2_ dielectric layers [9, 10].

Nowadays, PDI_CY n-type OFET, exhibiting charge carrier mobility higher than 1 cm^2^/Volt sec [11, 12] are among the most studied devices in the emerging field of organic electronics [5]*.* PDI_CY semiconducting films exhibit also a strong interaction with light and the electrical response of the related devices can be so largely modified through the illumination by visible radiation [13].

Despite these appealing properties, up to date there is a substantial lack of experimental studies focalized on the charge transport properties of the PDI_CY molecules at the nanoscale level. In particular, the electrical behavior of single molecular PDI_CY junctions has never been investigated, different from what reported for other PDI compounds, functionalized with thiol or pyridil anchoring groups at imide side positions [14], which have been characterized even in three-terminal single-molecule devices displaying intriguing electronic functionalities [15, 16]. To this purpose, it is to be outlined that the electrical testing of single-molecule PDI_CY devices should be inherently favored by the presence of the cyano moieties, playing the role of anchoring groups with respect to gold nano-electrodes [17]. As reported also in another recent study focalized on synchrotron-based experiments, indeed, a strong chemical interaction, basically mediated by the presence of the cyano groups, takes place between PDI_CY molecules and Au surface [18].

Beyond the perspective to develop nanoscale or single-molecule devices with robust electronic functionalities, the study of PDI_CY molecular junctions could offer significant contributions also in elucidating some fundamental and still unclear aspects concerning the electrical response of devices based on large solid-state assemblies of these molecules. In particular, variations at the nanoscale level in the bonding configurations between molecules and electrode atoms could lead to not negligible fluctuations in the local electronic structure of the metal-organic interfaces [19]. As widely recognized, this interfacial energetics plays a fundamental role in the charge-injection basic phenomena which, for many organic devices, can strongly affect the overall electrical response.

In this paper, the conductance properties of single PDI_CY molecules are studied by using mechanically controllable break junctions (MCBJ) employing gold nano-electrodes. MCBJ technique represents a fundamental approach to study the electronic properties of single molecules, offering a continuously tunable gap size between two nanometer-sized metallic electrodes with picometer resolution, high mechanical stability of the system, and reduction of contaminants [20–22]. Through MCBJ experiments, single-molecule conductances of several compounds, ranging from dielectric alkanes via π-conjugated molecular wires to organic conductors like oligothiophenes, were recently investigated [23–25]. In this work, single molecular junctions were fabricated considering N,N0–bis(n-octyl)-1,6-dicyanoperylene-3,4:9,10-bis(dicarboximide (PDI8-CN_2_) molecules. A large series of conductance curves was collected and converted into one- and two-dimensional conductance histograms. From a statistical analysis of our experimental data, we extract the most probable conductance of the PDI8-CN_2_ single molecular junctions. The experimental observations are then discussed in the light of density functional theory (DFT) combined with non-equilibrium Green’s function (NEGF) calculations, estimating the transmission coefficients trough different paths for a PDI8-CN_2_ molecule in gas phase.

## Methods

**Fig. 1:**
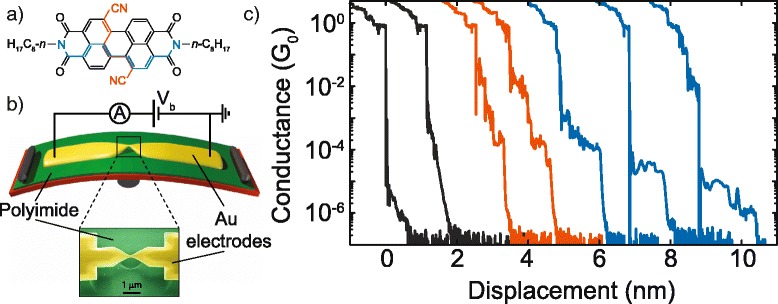
Experimental method. **a** Molecular structure of PDI8-CN_2_. **b** Scheme of the mechanically controlled break-junction set-up. *Inset*, colorized scanning electron microscopy (SEM) image of the central gold constriction. **c** Individual breaking traces of molecular junctions after the deposition of a droplet of the PDI8-CN_2_/dichloromethane solution onto the MCBJ device. For sake of clarity, the *curves* were shifted on the *x*-axis

PDI8-CN_2_ (see Fig. 1a for the molecular structure) was purchased from the POLYERA company and used without any further purification. The MCBJ devices were fabricated by patterning a gold wire by electron-beam lithography on top of a flexible phosphorous bronze substrate, coated with an insulating layer of polyimide. The nanowire central region consists of a 40 nm-wide constriction, suspended on the substrate after the etching of the polyimide by reactive ion etching in O_2_-CF_4_ plasma. More details on the MCBJ fabrication process can be found in [19, 18]. The MCBJ overall structure is schematically shown in Fig. 1b.

During the electrical characterization, the flexible substrate was clamped between two lateral supports and its central part was put in contact with a pushing rod, driven by a piezoelectric system. When the substrate was bent, the gold nanowire was stretched until its rupture and two nano-sized electrodes were formed whose separation can be adjusted mechanically. The technique consists of repeatedly moving the two metallic nano-electrodes into and out of contact. After the introduction of molecules into the gap, these may bridge the two electrodes and their electronic properties can be determined for different values of the electrode separation.

Statistical analysis allowed us to obtain the most probable conductance values for a single-molecule junction by collecting thousands of breaking curves (see Fig. 1c) and building conductance histograms. In this work, the conductance of the MBCJ devices was measured at room temperature applying a bias voltage of 0.1 V and measuring the related current (sampling rate of 2 KHz) by a battery-powered logarithmic amplifier capable of recording currents over a range of nine orders of magnitude. During the electrical tests, the pushing rod was moved at the speed of 4 nm/s. To improve the reproducibility of our results, different samples were considered during various days.

Concerning calculations, we used the Amsterdam Density Functional (ADF) quantum chemistry package [26, 27]. The calculations were performed using a triple-ζ plus polarization (TZP), Slater-type orbital local basis-set in the Perdew-Burke-Ernzerhof parameterization of the generalized gradient approximation functional (GGA-PBE) [28]. Using the NEGF formalism, the molecule was connected to wide-band limit contacts, and the transmission was calculated [29].

## Results and Discussion

For the realization of the molecular break junctions, PDI8-CN_2_ was first dissolved (1 mmol concentration) in dichloromethane at room temperature and, then, the molecules were deposited by pipetting a 2 μL droplet of this solution on the MBCJ devices.

A representative set of conductance versus displacement curves, recorded while separating the electrodes, is reported in Fig. 1c. As shown, all these experimental curves exhibit a plateau in conductance at a value close to 1 G_0_ (G_0_ = 2e^2^/h ~77.5 μS), the quantum of conductance. This occurrence confirms the formation of a single Au-Au bond bridging the gap between the two MCBJ electrodes. Some of the recorded breaking curves (the black ones in Fig. 1c) did not display any signature related to the presence of PDI8-CN_2_ molecules. In this case, indeed, we observe only through-space tunneling between the metallic electrodes, with the measured conductance decreasing exponentially as a function of the electrode displacement. The lack of counts between 1 and 10^−3^ G_0_ has been assigned to the fast retraction of the first gold atoms after the breaking of the nanowire (jump out-of-contact) [30]. It should be also outlined that the noise level for the considered experimental set-up was estimated lower than 2 · 10^−7^ G_0_ (see the noise in the conductance present in all the traces around 2 10^−7^ G_0_ in Fig. 1c).

For other breaking curves (the orange ones in Fig. 1c), the formation of junctions with a PDI8-CN_2_ molecule actually bridging the two nano-electrodes was identified by the observation of conductance (G) plateaus mainly located in the range between 10^−2^ and 10^−3^ G_0_ and characterized by a length of around 1 nm. Some of these curves also showed a slight and continuous decrease in conductance as a function of the stretching distance, followed by a steep drop occurring at about 10^−4^ G_0_.

Finally, another set of breaking curves (the blue ones in Fig. 1c) displayed G plateaus ranging from 10^−4^ to 10^−6^ G_0_. In this case, the plateau length in terms of the electrode displacement was in average larger (from 1 to 1.5 nm) than that observed for the high-G plateaus before discussed

**Fig. 2:**
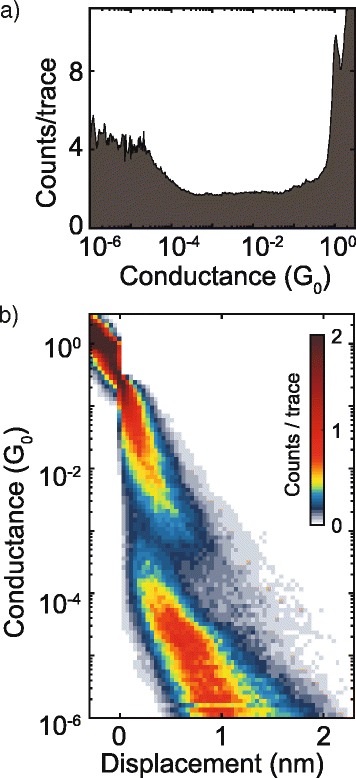
Conductance histograms. **a** One-dimensional and **b** two-dimensional conductance histograms built from 2656 traces recorded for PDI8-CN_2_-based MCBJ devices. The *curves* are binned with 30 bins/nm on the *x*-axis, and 31 bins/decade on the *y*-axis. The *color scale* in the trace histogram indicates the density of data points found at each displacement and conductance value; in this way, the *red areas* represent the most probable evolution during the breaking process

In order to get a more quantitative description of the main features above identified, several thousands of individual breaking traces were recorded. Then, they were further analyzed by constructing conductance histograms to extract statistically significant results as a function of the electrode displacement values. Figure 2a reports the corresponding one-dimensional (1D) histogram, built from 2656 consecutive breaking curves.

Here, the peak appearing at 1 G_0_, due to single gold atom contacts, is a sign of atomically sharp contacts. Below 1 G_0_, no clear peaks are present in the range between 10^−2^ G_0_ and 10^−4^ G_0_, while, instead, an increase of counts between 10^−4^ G_0_ and 10^−6^ G_0_ appears evident. This last feature could be related to a superimposition effect between counts associated to the tunneling phenomenon between the nano-electrodes and counts ascribable to the low-conductance molecular paths.

A more meaningful description about the overall set of the recorded breaking curves was gained by the construction of a two-dimensional conductance histogram, where the number of counts is plotted for any specific conductance/electrode displacement combination (Fig. 2b). This 2D graph was achieved by aligning all individual conductance traces to a common zero displacement reference point, taken at the rupture of the gold metallic contact. In the two-dimensional histogram, besides the clear region of high counts around 1G_0_ , it is also well distinguishable an area (mainly yellow colored) of high counts for conductance values between 10^−2^ and 10^−3^ G_0_ and electrode displacement ranging between 0.5 and 1 nm.

**Fig. 3:**
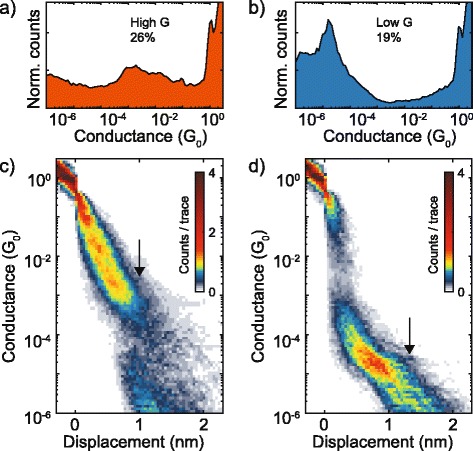
High and low-conductance configurations. One-dimensional and two-dimensional conductance histograms extracted for high conductance (**a**, **c**) and low conductance (**b**, **d**), respectively. The most probable conductance values were extracted by fitting the peaks of the 1D-conductance histograms to a Gaussian function (see Additional file 1)

The second region exhibiting high counts (mainly red colored), instead, extends over the electrode displacement region between 1 and 2 nm and is due to the partial overlap between the tunneling phenomenon (due to the empty junctions) and the molecular paths displaying G values lower than 10^−4^ G_0_. In order to estimate the most probable conductance values of single PDI8-CN_2_ molecules, conditional 1D- and 2D-histograms, achieved from the selection of individual curves showing only high-G or low-G plateaus, were constructed (Fig. 3).

The 1D-histogram in Fig. 3a was utilized to extract the most probable high-G value by fitting the broad peak in the region between 10^−2^ and 10^−3^ G_0_ to a Gaussian function (see Additional file 1). The best fit was so obtained for a Gaussian centered at the value of 2.5 10^−3^ G_0_. On the other hand, the histogram in Fig. 3b displays instead a very well defined peak located at 1.8 10^−5^ G_0,_ which can be consequently taken directly as the most probable value for the low-G-region. The related 2D-histogram (Fig. 3d), where the tunneling phenomenon contributions have been excluded, makes also clear that the low-G molecular path is centered at about 1 nm.

It is quite significant to remember that recent experiments, performed through the STM-based break junction approach on other perylene-diimide derivatives with different functionalization in the bay or side aromatic regions, reveal the presence for all these compounds of only one most probable molecule junction conductance value which, in any case, never exceeds 10^−4^ G_0_ [13, 11].

**Fig. 4:**
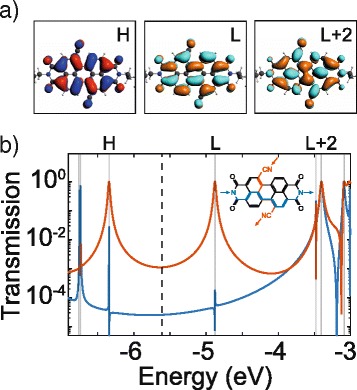
DFT+NEGF calculations on the PDI8-CN_2_ molecule. **a** Frontier orbitals of a PDI8-CN_2_ molecule in gas phase calculated from DFT. HOMO, LUMO and LUMO+2 are called, respectively, as H, L, and L+2. **b** NEGF calculations of the transmission through N-to-N atoms of the cyano groups (*red curve*) and N-to-N atoms located at the side positions (*blue curve*). The *dashed line* indicates the mid-gap energy between H and L

To gain better insight in the transport mechanism of the perylene molecular junctions, we model the charge transport in the molecule. Theoretically, the transport of charge through a molecule can be described as a transmission between the different molecular orbitals in the Landauer formalism. We have calculated the transmission through the PDI8-CN_2_ molecule using the NEGF method with a DFT calculation of the ground-state electron density. In Fig. 4a, we show the calculated charge density plot of the π-like frontier orbitals HOMO, LUMO, and LUMO+2 calculated from DFT. We notice that HOMO-1 and HOMO-2 as well as LUMO+1 are σ-orbitals, and thus do not contribute significantly to the transport. In Fig. 4b, we plot the calculated transmission through the π-system in the wide-band limit for two different charge-injection cases that simulate two possible molecule-electrode configurations. In one case, the charge is injected in the nitrogen atoms (N1) of the cyano groups (path 1, red curve), and in the other case, the charge is injected in the pyridil-like terminal (N2) nitrogen atoms (path 2, blue curve), as shown schematically in the inset of Fig. 4b.

At energies between the HOMO (−6.6 eV) and LUMO (−5.1 eV) energies, the transmission through path 1 is much larger than through path 2. Thus, the injection of the charge in the N1 atoms provides an electrical path that is around two orders of magnitude less resistive than the path through the other couple of N2 atoms. The length of path 1 and of path 2, measured from nitrogen to nitrogen, is 0.95 nm in the first case and 1.13 nm in the second case and this difference alone cannot explain the large difference between the conductance of the two paths. Instead, the reason for the large conductance change can be found in the shape of the molecular orbitals extracted from DFT (Fig. 4a). When inspecting the frontier orbitals HOMO and LUMO, one sees that on the cyano nitrogen atoms there is some finite density of states, while, at the positions of N2 nitrogen atoms, a node in the wave-function appears, making the effective density of states on those atoms zero. To recover some density of π-states on the terminal N2 atoms, one has to look for the LUMO+2 and HOMO-5 orbitals. The larger effective transport gap of path 2 (HOMO-5/LUMO+2) in respect to the normal gap of path 1 (HOMO/LUMO) can explain the difference in conductance between the two configurations.

Thus, DFT and NEGF calculations of the PDI8-CN_2_ molecule indicate the presence of two possible electrical paths through the molecule. A shorter and more conducting path, related to the cyano groups, and a longer and more resistive path, through the terminal (N2) nitrogen atoms. The ratio between the conductance of the two paths and the difference in length are comparable with the two experimental conductance paths, found from the histogram analysis of Fig. 3. At this level of theory, a quantitative agreement with the experiments is not expected and can be coincidental. Nevertheless, the experimental ratio (*R*_EXP_ = 130) between high and low-conductance paths is very similar to the theoretical ratio between the conductance of path 1 and 2. The experiment length of the traces is also comparable with the length of the two paths found from theory (see Additional file 1). We thus assign the experimental high conductance traces to junctions where the molecule interacts with the metal through the cyano groups. The low-conductance traces, instead, can be assigned to the conduction through N2 atoms (path 2).

In this regard, it is quite interesting to outline that recent experiments involving molecular junctions based on different PDI compounds, without the functionalization of the cyano groups in the bay region, have demonstrated the occurrence of only one most probable conductance state with G values never exceeding 10^−4^ G_0_ [11, 13]. In particular, for PDI compounds terminated with pyridyl anchoring groups, most probable junction conductance values are quite close to that here estimated for the molecular path involving the terminal nitrogen (N2) atoms [11]. This comparison seems to give a further confirmation about the role of the CN groups in the introduction of charge transport paths with improved conducting features through the single molecules.

## Conclusions

Mechanically controllable break junction experiments and DFT calculations performed in this work suggest that the functionalization through the insertion of cyano (C≡N) in the basic perylene-diimide aromatic core is able to determine a preferential charge transport molecular path exhibiting a high conductance value comprised between 10^−2^ e 10^−3^ G_0_. This value is about two orders of magnitude larger than that can be achieved by considering instead a path linking the pyridil-like terminal (N2) nitrogen atoms. The low-G value here observed is compatible with single molecule conductances recently reported for other perylene-diimide compounds in absence of the cyano functionalization. Future experiments on PDI_CY single molecular junctions are envisaged to confirm the interest of this class of compounds to be applied in the realization of nanoscale devices.

## Additional file

Additional file 1:
**Single-molecule break junctions based on a perylene-diimide cyano-functionalized (PDI8-CN**_**2**_ **) derivative.**
