# Supplementary material for: Single-Molecule Break Junctions Based on a Perylene-Diimide Cyano-Functionalized (PDI8-CN2) Derivative
Source: Nanoscale Res Lett. 2015 Jul 28;10:305. doi: 10.1186/s11671-015-1011-3 (PMC4516147; doi:10.1186/s11671-015-1011-3)
Supplement: Additional file 1: — Single-molecule break junctions based on a perylene-diimide cyano-functionalized (PDI8-CN 2 ) derivative. [file 11671_2015_1011_MOESM1_ESM.doc]

**Supporting information for**

Single–molecule break junctions based on a perylene-diimide cyano functionalized (PDI8-CN_2_) derivative

Riccardo Frisenda^1^, Loredana Parlato^2^, Mario Barra^2^, Herre S. J. van der Zant^1^, Antonio Cassinese^2^

^1^ Kavli Institute of Nanonscience, Delft University of Technology, Lorentzweg 1 2628 CJ, Delft, The Netherlands

^2^CNR-SPIN and Physics Department, University of Naples, Piazzale Tecchio 80, I-80125 Naples, Italy

**Transport measurements**


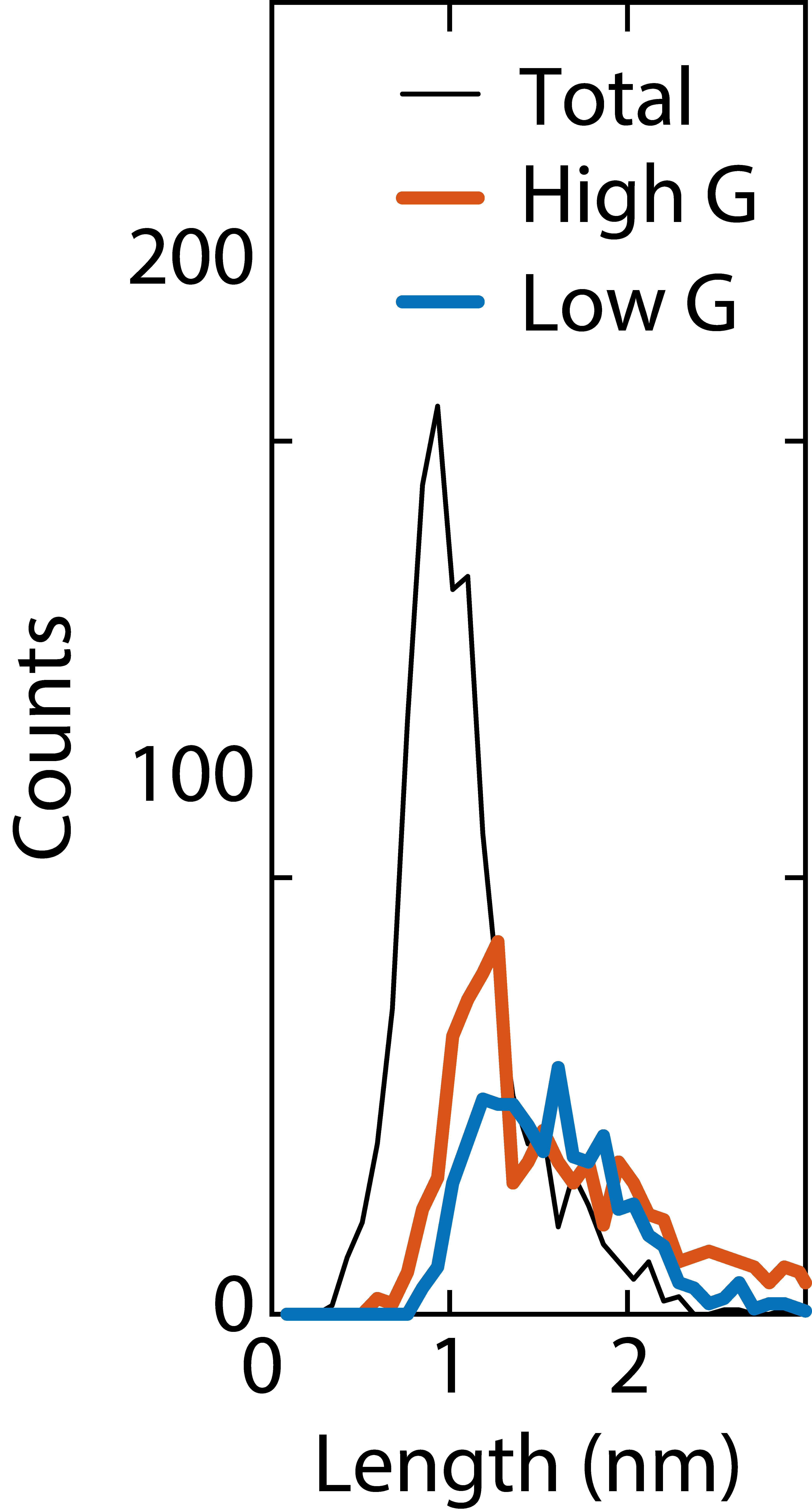


Figure S1 Length histograms built respectively from all the individual 2656 conductance traces (black line) measured in presence of PDI8-CN_2_, and from traces selected to contain high G plateaus (red line) and low G plateaus (blue line). The length of each individual trace is defined as the distance that it takes for the conductance to go from 10^-1^ G_0_ to 10^-6^ G_0_.


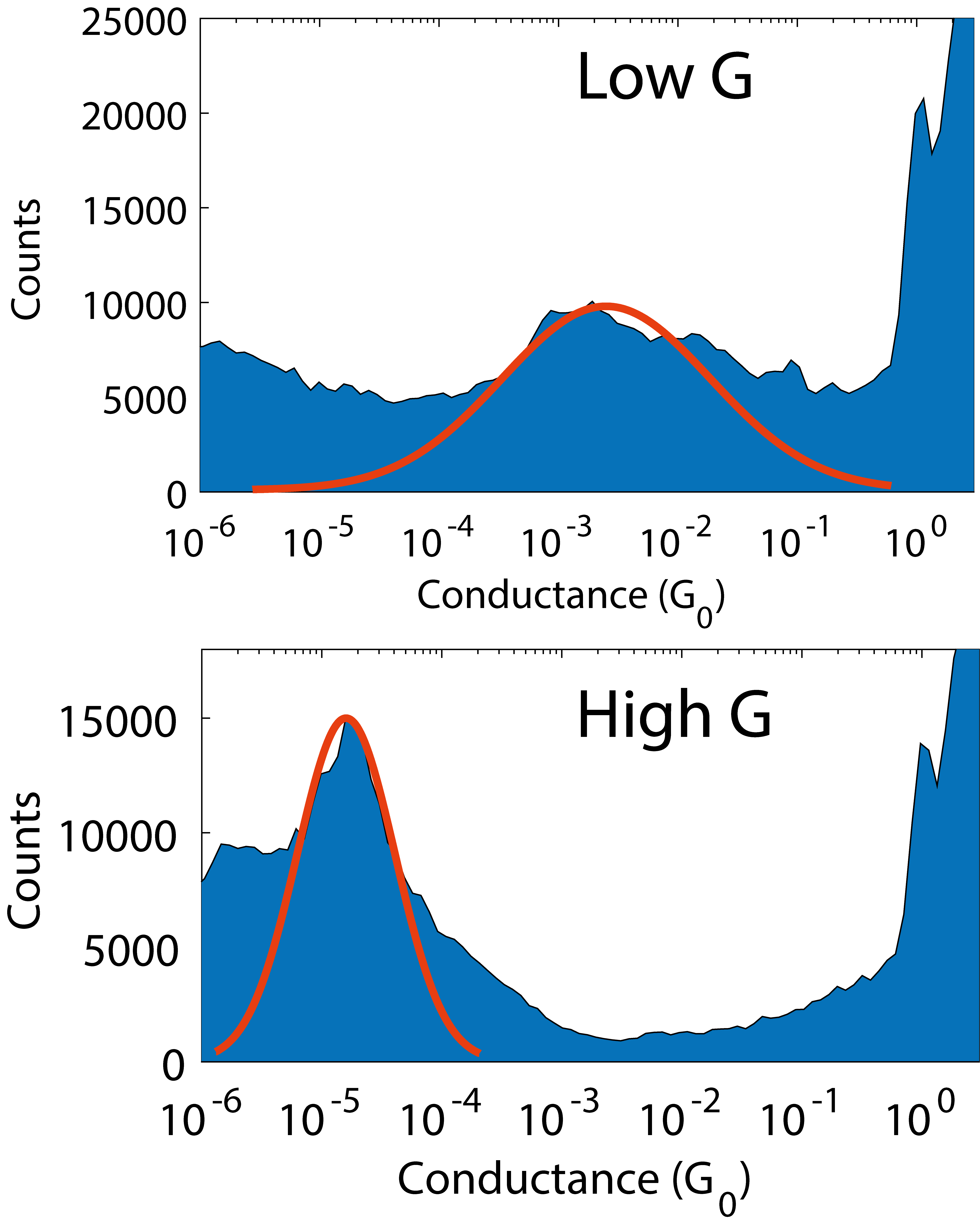


**High G**

**Low G**

Figure S2 One-dimensional conductance histograms extracted for high conductance (top) and low conductance (bottom), respectively. The most probable conductance values were extracted by fitting the peaks of the 1-D-conductance histograms to a Gaussian function (red lines).


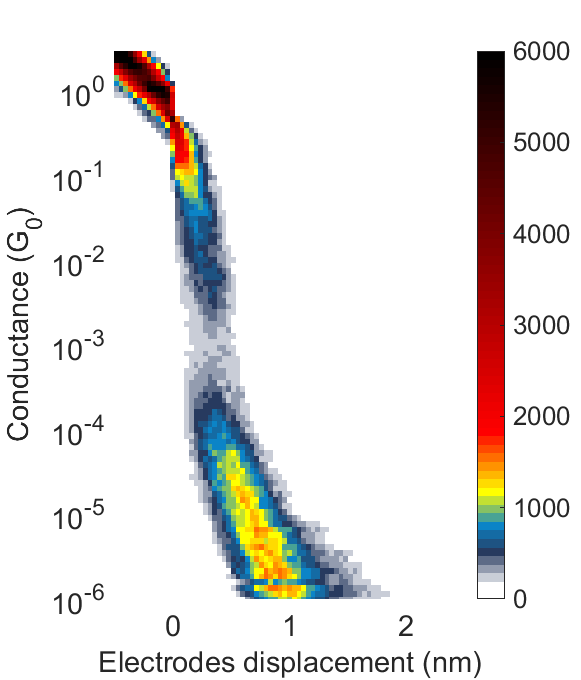


Figure S3 Two-dimensional conductance histogram built from all the traces not showing high G or low G plateaus.
